# Detection of *Aspergillus* in the lower respiratory tract and its association with mortality in patients with different viral pneumonias: a retrospective multicenter cohort study from China

**DOI:** 10.3389/fcimb.2026.1906021

**Published:** 2026-09-22

**Authors:** Xiaohua Li, Xinwei He, Xufeng Fu, Nan Wu, Deqing Yang, Jin Zhao, Xinqian Ma, Shukun Chai, Wentao Ni

**Affiliations:** 1 Department of Respiratory and Critical Care Medicine, Fuzhou First General Hospital Affiliated with Fujian Medical University, Fuzhou, China; 2 Department of Pulmonary and Critical Care Medicine, Fujian Medical University Union Hospital, Fuzhou, China; 3 Department of Internal Medicine, Xicheng District Zhanlanlu Hospital, Beijing, China; 4 Department of Respiratory and Critical Care Medicine, The Seventh Affiliated Hospital of Sun Yat-sen University, Shenzhen, China; 5 Department of Respiratory and Critical Care Medicine, Peking University People’s Hospital, Beijing, China; 6 Department of Pharmacy, The Second Affiliated Hospital of Kunming Medical University, Kunming, China; 7 Department of Respiratory Diseases, Air Force Medical Center, Chinese People’s Liberation, Beijing, China; 8 Department of Pulmonary and Critical Care Medicine, Shijiazhuang People’s Hospital, Hebei, China

**Keywords:** *Aspergillus*, cohort study, influenza, mortality, SARS-CoV-2, viral pneumonia

## Abstract

**Background:**

The detection of *Aspergillus* in the lower respiratory tract complicates the clinical course of viral pneumonia and is linked to poor outcomes, but prior evidence is confined to the intensive care unit (ICU) or single-virus settings (e.g., influenza or SARS-CoV-2). Whether its frequency and prognostic significance differ across viral etiologies in hospitalized adults remains unknown.

**Methods:**

This multicenter retrospective cohort study included adults with community-acquired viral pneumonia at seven tertiary hospitals in China (2017-2025). Patients were categorized into SARS-CoV-2, influenza, or other viral groups. The exposure was lower respiratory tract *Aspergillus* detection (via using culture, real-time PCR, or metagenomic next-generation sequencing). The primary outcome was in-hospital all-cause mortality.

**Results:**

Among 929 patients, *Aspergillus* was detected in 175 (18.8%). The detection rates differed significantly across viral etiologies (33.2% in patients with influenza, 14.6% in those with SARS-CoV-2, and 7.4% in those with other viruses; P < 0.001). In the fully adjusted model, *Aspergillus* detection was independently associated with increased overall mortality (aOR = 2.99; 95% CI 1.60–5.58; P < 0.001), but the association varied markedly by viral etiology. Virus-stratified analyses revealed that in influenza, *Aspergillus* detection remained independently associated with mortality after multivariable adjustment, and the association was consistently observed across prespecified subgroups, including non-ventilated patients, whereas in SARS-CoV-2, the association was largely confined to ventilated patients.

**Conclusion:**

Lower respiratory tract *Aspergillus* detection differed substantially by viral etiology and was most frequent in patients with influenza pneumonia. It was independently associated with increased mortality across all influenza subgroups, including non-ventilated patients, whereas in SARS-CoV-2 pneumonia this association was largely confined to mechanical ventilation patients. These findings support a virus-stratified approach to fungal surveillance and warrant prospective validation.

## 1 Introduction

Viral pneumonia is a major cause of hospitalization, acute respiratory failure, and mortality in adults, particularly among older adults and those with chronic comorbidities (Jain et al., 2015; Hanage and Schaffner, 2025). Over the past decade, seasonal and pandemic influenza, SARS-CoV-2, and other viruses have collectively altered the epidemiology and clinical presentation of viral pneumonia (Chow et al., 2023; Cilloniz et al., 2025). Patients with viral pneumonia are susceptible to various secondary infections, among which fungal infections—particularly invasive pulmonary aspergillosis (IPA)—have emerged as important complications (Schauwvlieghe et al., 2018; Magira et al., 2019; MaChado et al., 2021).

Respiratory viruses can damage airway epithelium, impair mucociliary clearance, and disrupt innate and adaptive immunity, thereby weakening antifungal defenses (Bridges et al., 2022; Salazar et al., 2022; Nguyen et al., 2024; Seldeslachts et al., 2024; Horton et al., 2025; Xiang et al., 2025). Moreover, high-dose or prolonged glucocorticoid use, broad-spectrum antibiotics, and mechanical ventilation are recognized risk factors for IPA (Montrucchio et al., 2021; Hurt et al., 2023; Terrones-Campos et al., 2026). In intensive care unit (ICU) cohorts, influenza-associated invasive pulmonary aspergillosis (IAPA) has been reported in approximately 19%–29% of patients, leading to prolonged ICU stays and mortality often exceeding 45% (Chong et al., 2022; Lu et al., 2024). In contrast, COVID-19-associated pulmonary aspergillosis (CAPA) affected approximately 2%–25% of critically ill patients, with ICU mortality exceeding 50% (Prattes et al., 2022). However, most previous studies have focused on highly selected ICU populations, primarily addressing IAPA or CAPA, with small sample sizes and few systematic comparisons between viral etiologies.

In real-world hospital settings, the detection of *Aspergillus* in the lower respiratory tract—which often represents colonization or early infection rather than confirmed IPA—is a frequent finding among patients with viral pneumonia. Notably, our previous work demonstrated that such early detection is associated with higher mortality in influenza pneumonia (Yin et al., 2023). Whether the rate of *Aspergillus* detection and its prognostic implications differ by viral etiology (SARS-CoV-2, influenza, or other viruses) remains unknown in real-world hospitalized populations. To address this question, we conducted a multicenter retrospective cohort study of adult inpatients with community-acquired viral pneumonia to compare *Aspergillus* detection rates across different viral etiologies and to evaluate whether the associated mortality risk varies depending on the causative virus.

## 2 Materials and methods

### 2.1 Ethics approval

The study protocol was approved by the Ethics Committee of Shijiazhuang People’s Hospital (Approval No. 2026090) and conducted in accordance with the Declaration of Helsinki. Given the retrospective design and use of anonymized data, the Ethics Committee waived the requirement for informed consent.

### 2.2 Study participants

We included patients admitted to seven tertiary hospitals in China with viral pneumonia between February 1, 2017, and April 1, 2025, reported as per STROBE guidelines (von Elm et al., 2007).

Inclusion criteria: (1) diagnosis of community-acquired pneumonia using standard criteria (clinical signs and symptoms plus radiographic evidence of alveolar inflammation, per the 2025 ATS guideline (Jones et al., 2026); (2) laboratory confirmation of viral infection. This confirmation required either a positive result for viral pathogens (including SARS-CoV-2, influenza A/B, adenovirus, herpes simplex virus, rhinovirus, parainfluenza, human coronavirus, respiratory syncytial virus, cytomegalovirus, coxsackievirus, human metapneumovirus, and varicella-zoster virus) via real-time PCR or metagenomic next-generation sequencing of respiratory specimens (sputum, tracheal aspirates, bronchoalveolar lavage fluid, or nasopharyngeal swabs), or a positive rapid antigen test for SARS-CoV-2 or influenza.

Patients were excluded if they met any of the following criteria: hospitalization within 30 days prior to admission; pulmonary fungal infection within 3 months prior to admission; pulmonary embolism; NYHA class III-IV heart failure; active tuberculosis or nontuberculous mycobacterial infection; HIV infection; pregnancy or lactation; or mixed viral infection.

### 2.3 Data collection

Clinical data were systematically extracted from the electronic medical records of all seven participating centers and entered into a standardized case report form via the Free Electronic Data Capture system (version 2.0; Beijing Free Clinical Medical Technology Co., Ltd., Beijing, China). Data extraction was performed independently by two trained clinical research coordinators at each site. Any discrepancies were resolved through discussion or by adjudication from the principal investigator.

The following data domains were collected for each patient: (1) Demographic and baseline characteristics, including age, sex, and smoking history; (2) Comorbidities, including: hypertension, diabetes mellitus, coronary heart disease (CHD), cerebrovascular disease, chronic lung disease (asthma, chronic obstructive pulmonary disease, or diffuse interstitial lung disease), chronic kidney disease (CKD) (with or without dialysis), chronic liver disease (hepatitis, cirrhosis, or liver failure), and immunosuppression (attributable to underlying autoimmune disease, long-term immunosuppressive medication, or active malignancy); (3) Vital signs at hospital admission, including: maximum body temperature (T_max_), pulse rate, systolic and diastolic blood pressure, and respiratory rate; (4) Laboratory parameters obtained within 24 h of admission, including: complete blood count [white blood cell (WBC) count, neutrophil (NEU) count, lymphocyte (LYM) count, monocyte count, hemoglobin (HGB), and platelet (PLT) count], liver function tests (alanine aminotransferase; aspartate aminotransferase and lactate dehydrogenase), creatine kinase, serum albumin (ALB), serum creatinine, prothrombin time, D-dimer, C-reactive protein (CRP), and procalcitonin (PCT); and arterial blood gas analysis (pH, arterial partial pressure of carbon dioxide, arterial partial pressure of oxygen, oxygenation index [arterial partial pressure of oxygen/fraction of inspired oxygen], and lactic acid (Lac)]; (5) Microbiological data, specifically: the results of *Aspergillus* detection in lower respiratory tract samples (sputum, tracheal aspirate, or bronchoalveolar lavage fluid) via fungal culture, real-time PCR, or metagenomic next-generation sequencing. Sputum specimens were subjected to cytological quality screening prior to processing; eligibility was defined as fewer than 10 squamous epithelial cells and more than 25 leukocytes per low-power field. (6) Treatment data, including antiviral, antibacterial, and antifungal therapies, systemic glucocorticoid use, and invasive mechanical ventilation; and (7) clinical outcomes, specifically in-hospital all-cause mortality, and 28-day in-hospital mortality was also assessed as a secondary outcome.

### 2.4 Statistical analysis

Statistical analyses were performed using R version 3.6.3 and Free Statistics version 2.2. Missing laboratory and clinical values (e.g., D-dimer, albumin) were imputed using multiple imputation by chained equations (MICE; 20 imputed datasets); the proportions of missing data are summarized in **Supplementary Table S1**. Continuous variables are presented as means ± standard deviations (SD) or medians with interquartile ranges (IQR), and were compared using analysis of variance (ANOVA) or the Kruskal-Wallis test, as appropriate. Categorical variables are expressed as frequencies (percentages) and were compared using the Pearson χ² or Fisher’s exact test. Univariate logistic regression models were used to estimate odds ratios (ORs) and 95% confidence intervals (CIs) for *Aspergillus* detection and in-hospital mortality. Variables with P < 0.05 in the univariate analysis, alongside clinically relevant confounders, were incorporated into sequentially built multivariable models: Model 1 adjusted for demographics (age, sex, and smoking status); Model 2 further adjusted for inflammatory markers and laboratory findings; Model 3 additionally included comorbidities; and Model 4 (the full model) further incorporated antiviral and antibacterial therapies, systemic glucocorticoids, as well as mechanical ventilation. Pre-specified stratified analyses were performed to assess effect modification by age (<60 vs. ≥60 years), sex, presence of comorbidities, and mechanical ventilation in the overall cohort. In addition, we conducted subgroup analyses within the influenza and SARS-CoV-2 subgroups to assess the moderating effects of age, sex, and mechanical ventilation on the effect. Interactions were assessed by including multiplicative interaction terms in the logistic regression models, and statistical significance was determined by likelihood ratio tests. A two-sided P < 0.05 was considered statistically significant.

## 3 Results

### 3.1 Study flow and pathogen detection results

Of the 962 screened patients with confirmed viral pneumonia (**Figure 1**), 929 were included in the final analysis after excluding 3 patients aged <18 years and 30 with missing data for key categorical variables; the viral-type distribution is shown in **Supplementary Figure S1**.

**Figure 1:**
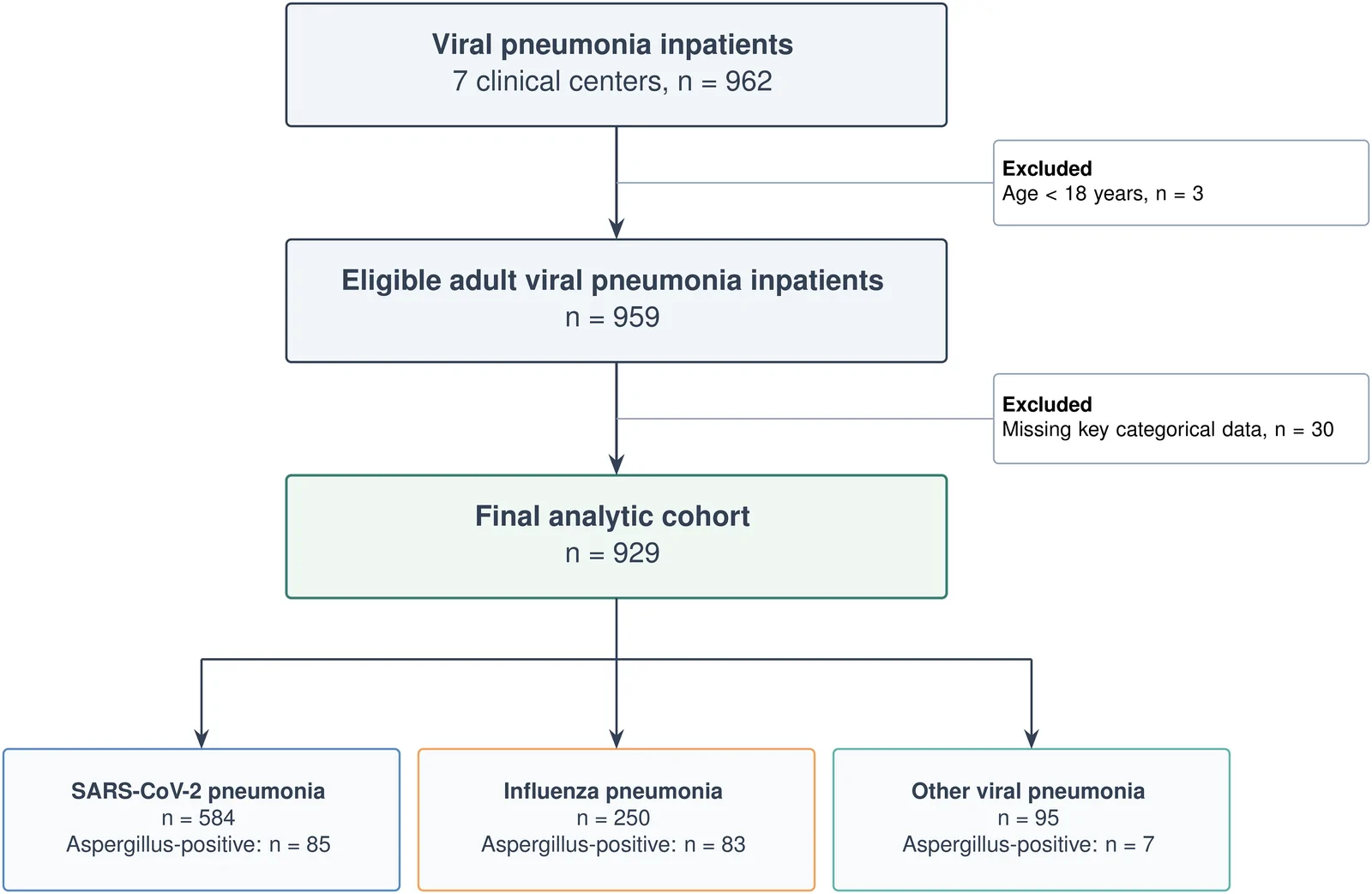
Study flow chart.

Baseline characteristics by virus type are summarized in **Table 1**, revealing significant differences in demographics, comorbidities, laboratory parameters, and treatments (further results in **Supplementary Table S2**). Patients in the SARS-CoV-2 group were the oldest (75.6 ± 11.5 years), and had the longest hospital stay (25.5 ± 9.9 days). This group also exhibited the highest prevalences of hypertension, CHD, and cerebrovascular disease, alongside the lowest ALB and LYM counts, and the highest rate of glucocorticoid use (66.3%). Conversely, chronic lung disease was most common in the influenza group (29.6%), which also demonstrated the highest D-dimer levels and the highest frequencies of antiviral (75.2%) and antifungal (36.0%) therapies. Meanwhile, a history of smoking was most prevalent among patients infected with other viruses (45.3%).

**Table 1:** Baseline clinical characteristics, treatments, and outcomes of patients stratified by viral pneumonia type.

Variables	Total (n = 929)	SARS-CoV-2 (n = 584)	Flu (n = 250)	Other viruses (n = 95)	P	Statistic
Aspergillus detection, n (%)	175 (18.8)	85 (14.6)	83 (33.2)	7 (7.4)	< 0.001	48.91
Baseline characteristics						
Sex, n (%)					0.49	1.426
Male	612 (65.9)	388 (66.4)	158 (63.2)	66 (69.5)		
Female	317 (34.1)	196 (33.6)	92 (36.8)	29 (30.5)		
Age, Mean ± SD	71.2 ± 14.8	75.6 ± 11.5	64.9 ± 16.0	60.9 ± 18.1	< 0.001	85.199
Smoking history, n (%)	267 (28.7)	133 (22.8)	91 (36.4)	43 (45.3)	< 0.001	29.976
Length of stay (days), Mean ± SD	21.3 ± 11.2	25.5 ± 9.9	14.5 ± 10.0	13.0 ± 8.0	< 0.001	148.431
Comorbidities						
Chronic lung diseases, n (%)	197 (21.2)	100 (17.1)	74 (29.6)	23 (24.2)	< 0.001	16.881
Hypertension, n (%)	487 (52.4)	346 (59.2)	114 (45.6)	27 (28.4)	< 0.001	37.512
Diabetes mellitus, n (%)	264 (28.4)	185 (31.7)	58 (23.2)	21 (22.1)	0.016	8.259
Coronary heart disease, n (%)	234 (25.2)	174 (29.8)	52 (20.8)	8 (8.4)	< 0.001	23.304
Cerebrovascular disease, n (%)	106 (11.4)	82 (14.0)	19 (7.6)	5 (5.3)	0.004	11.141
Chronic kidney disease, n (%)	71 (7.6)	45 (7.7)	20 (8.0)	6 (6.3)	0.867	0.285
Chronic liver disease, n (%)	42 (4.5)	31 (5.3)	11 (4.4)	0 (0)	0.035	Fisher
Immunosuppression, n (%)	117 (12.6)	65 (11.1)	36 (14.4)	16 (16.8)	0.18	3.435
Laboratory tests						
Lymphocyte count (×10^9^/L), Median (IQR)	0.9 (0.5, 1.3)	0.8 (0.5, 1.2)	0.8 (0.6, 1.4)	1.3 (0.9, 1.8)	< 0.001	48.866
Albumin (g/L), Mean ± SD	32.3 ± 6.5	31.6 ± 6.8	32.8 ± 5.9	35.6 ± 5.7	< 0.001	17.266
D-dimer (ng/mL), Median (IQR)	470.0 (190.0, 1270.0)	330.0 (100.0, 920.0)	698.6 (290.0, 1989.5)	823.0 (440.5, 1985.0)	< 0.001	65.289
Treatment						
Antiviral treatment, n (%)	523 (56.3)	325 (55.7)	188 (75.2)	10 (10.5)	< 0.001	117.299
Antibacterial treatment, n (%)	844 (90.9)	519 (88.9)	234 (93.6)	91 (95.8)	0.02	7.818
Antifungal treatment, n (%)	213 (22.9)	112 (19.2)	90 (36.0)	11 (11.6)	< 0.001	35.746
Glucocorticoid treatment, n (%)	462 (49.7)	387 (66.3)	59 (23.6)	16 (16.8)	< 0.001	173.268
Mechanical ventilation, n (%)	120 (12.9)	68 (11.6)	46 (18.4)	6 (6.3)	0.004	11.203
Clinical outcomes						
Death, n (%)	148 (15.9)	102 (17.5)	42 (16.8)	4 (4.2)	0.004	10.912

Data are presented as n (%), mean ± SD, or median (IQR). Chronic lung disease (asthma, chronic obstructive pulmonary disease, or diffuse interstitial lung disease), chronic heart disease (i.e., coronary artery disease or congestive heart failure, but not hypertension), immunosuppression (attributable to underlying autoimmune disease or long-term immunosuppressive medication, or active malignancy), diabetes, chronic kidney disease (with or without dialysis), chronic liver disease (hepatitis, cirrhosis, or liver failure); time of death refers to deaths occurring during hospitalization; Length of stay refers to the number of days from admission to discharge or death; any period of less than 24 hours is counted as one day.

Overall, *Aspergillus* was detected in lower respiratory tract specimens from 175 of the 929 patients (18.8%). By viral type, the detection rate was highest in the influenza group (83/250, 33.2%), followed by the SARS-CoV-2 group (85/584, 14.6%) and the other viruses group (7/95, 7.4%) (P < 0.001; **Table 1**). In-hospital mortality was significantly higher in the SARS-CoV-2 group (17.5%) and the influenza group (16.8%) than in the other virus groups (4.2%, P = 0.004). Additional laboratory test results and physical examination findings are provided in **Supplementary Table S2**.

### 3.2 Risk factors for *Aspergillus* detection

In univariate analysis, virus type was associated with *Aspergillus* detection (**Supplementary Table S3**): versus SARS-CoV-2, influenza carried a higher risk (OR = 2.92; 95% CI 2.06–4.14; P < 0.001), whereas other viruses did not differ significantly (OR = 0.47; 95% CI 0.21–1.04; P = 0.063). Other significant factors included chronic lung disease, CHD, chronic liver disease, glucocorticoids, and mechanical ventilation (ORs = 1.67–3.58, all P ≤ 0.016).

After sequential adjustment (Models 1–4), influenza remained associated with a higher risk of detection than SARS-CoV-2 (adjusted ORs = 2.66–3.29; all P < 0.001). Meanwhile, the other virus group exhibited a lower risk than the SARS-CoV-2 group, which remained statistically significant across Models 1–3 (**Table 2**).

**Table 2:** Multivariable-adjusted ORs and 95% CIs for the relationship between various viral infections and *Aspergillus* detection in the lower respiratory tract.

Variable	Total, n	Event, n(%)	Unadjusted univariate analysis		Model 1		Model 2		Model 3		Model 4	
			OR (95%CI)	P value	OR (95%CI)	P value	OR (95%CI)	P value	OR (95%CI)	P value	OR (95%CI)	P value
SARS-CoV-2	584	85 (14.6)	1(Ref)		1(Ref)		1(Ref)		1(Ref)		1(Ref)	
Flu	250	83 (33.2)	2.92 (2.06–4.14)	<0.001	2.82 (1.91–4.16)	<0.001	2.66 (1.78–3.96)	<0.001	2.69 (1.75–4.14)	<0.001	3.29 (1.88–5.74)	<0.001
Other viruses	95	7 (7.4)	0.47 (0.21–1.04)	0.063	0.40 (0.17–0.94)	0.036	0.34 (0.14–0.81)	0.015	0.39 (0.16–0.94)	0.037	1.12 (0.41–3.03)	0.828

Notes: Model 1 was adjusted for age, sex, and smoking. Model 2 was adjusted for Model 1 and WBC, NEU, D-dimer. Model 3 was adjusted for Model 2 and hypertension, CHD, diabetes, chronic lung disease, CKD, cerebrovascular disease, chronic liver disease, and immunosuppression. Model 4 was adjusted for Model 3 and antiviral drugs, antibacterial drugs, glucocorticoid and mechanical ventilation. WBC, white blood cell; NEU, neutrophil; CHD, coronary heart disease; CKD, chronic kidney disease.

In the stratified analysis (**Supplementary Figure S2**), age significantly modified this association (P for interaction = 0.008). Among patients aged ≥60 years, influenza was associated with a higher risk than SARS-CoV-2 (OR = 3.27; 95% CI 2.19–4.90; P < 0.001) while the risk in the other virus group did not differ significantly from that of SARS-CoV-2 (OR = 0.48; P = 0.167). Conversely, among those <60 years, the risk associated with influenza did not differ from that of SARS-CoV-2 (OR = 0.76; P = 0.507), but infection with other viruses was associated with a significantly lower risk (OR = 0.14; 95% CI 0.03–0.53; P = 0.004).

### 3.3 Association between *Aspergillus* detection and all-cause mortality

All-cause mortality was higher in *Aspergillus*-positive (33.1%) than negative (11.9%) patients (**Table 3**), and 28-day in-hospital mortality was consistently higher in *Aspergillus*-positive patients regardless of etiology (**Figure 2**).

**Table 3:** Multivariable-adjusted ORs and 95% CIs for the relationship between death and *Aspergillus* test results in viral pneumonia.

Different types of pneumonia	Variable	Total, n	Event, n (%)	Unadjusted univariate analysis		Model 1		Model 2		Model 3		Model 4	
				OR (95%CI)	P value	OR (95%CI)	P value	OR (95%CI)	P value	OR (95%CI)	P value	OR (95%CI)	P value
various types of viral pneumonia	*Aspergillus-*negative	754.0	90 (11.9)	1(Ref)		1(Ref)		1(Ref)		1(Ref)		1(Ref)	
	*Aspergillus*-positive	175.0	58 (33.1)	3.66 (2.49–5.37)	<0.001	4.54 (3–6.86)	<0.001	4.46 (2.82–7.06)	<0.001	4.05(2.48–6.61)	<0.001	2.99 (1.6–5.58)	<0.001
influenza pneumonia	*Aspergillus*-negative	167.0	15 (9)	1(Ref)		1(Ref)		1(Ref)		1(Ref)		1(Ref)	
	*Aspergillus*-positive	83.0	27 (32.5)	4.89 (2.42–9.85)	<0.001	4.79 (2.32–9.86)	<0.001	7.79 (3.09–19.62)	<0.001	5.78 (2.06–16.24)	0.001	13.77 (2.75–68.88)	0.001
SARS-CoV-2 pneumonia	*Aspergillus*-negative	499.0	72 (14.4)	1(Ref)		1(Ref)		1(Ref)		1(Ref)		1(Ref)	
	*Aspergillus*-positive	85.0	30 (35.3)	3.23 (1.94–5.39)	<0.001	4.54 (2.56–8.04)	<0.001	4.72 (2.43–9.18)	<0.001	4.49 (2.17–9.29)	<0.001	2.07 (0.77–5.55)	0.148
other viral pneumonia	*Aspergillus*-negative	88.0	3 (3.4)	1(Ref)		1(Ref)		1(Ref)		1(Ref)		1(Ref)	
	*Aspergillus*-positive	7.0	1 (14.3)	4.72 (0.42–52.57)	0.207	not estimable	1	not estimable	1	not estimable	1	not estimable	1

Model 1 was adjusted for age, sex, and smoking. Model 2 was adjusted for Model 1 and pulse rate, respiratory rate, potential of hydrogen, Lac, WBC, NEU, LYM, HGB, PLT, lactate dehydrogenase, ALB, CRP, and PCT. Model 3 was adjusted for Model 2 and hypertension, CHD, diabetes, chronic lung disease, CKD, cerebrovascular disease, chronic liver disease, and immunosuppression. Model 4 was adjusted for Model 3 and antiviral drugs, antibacterial drugs, glucocorticoids and mechanical ventilation. Adjusted models could not be reliably estimated due to small event numbers (n=7 with 1 event) in other viral pneumonia group. Lac, lactic acid; WBC, white blood cell; NEU, neutrophil; LYM, lymphocyte; HGB, hemoglobin; PLT, platelet; ALB, albumin; CRP, C-reactive protein; PCT, procalcitonin; CHD, coronary heart disease; CKD, chronic kidney disease.

**Figure 2:**
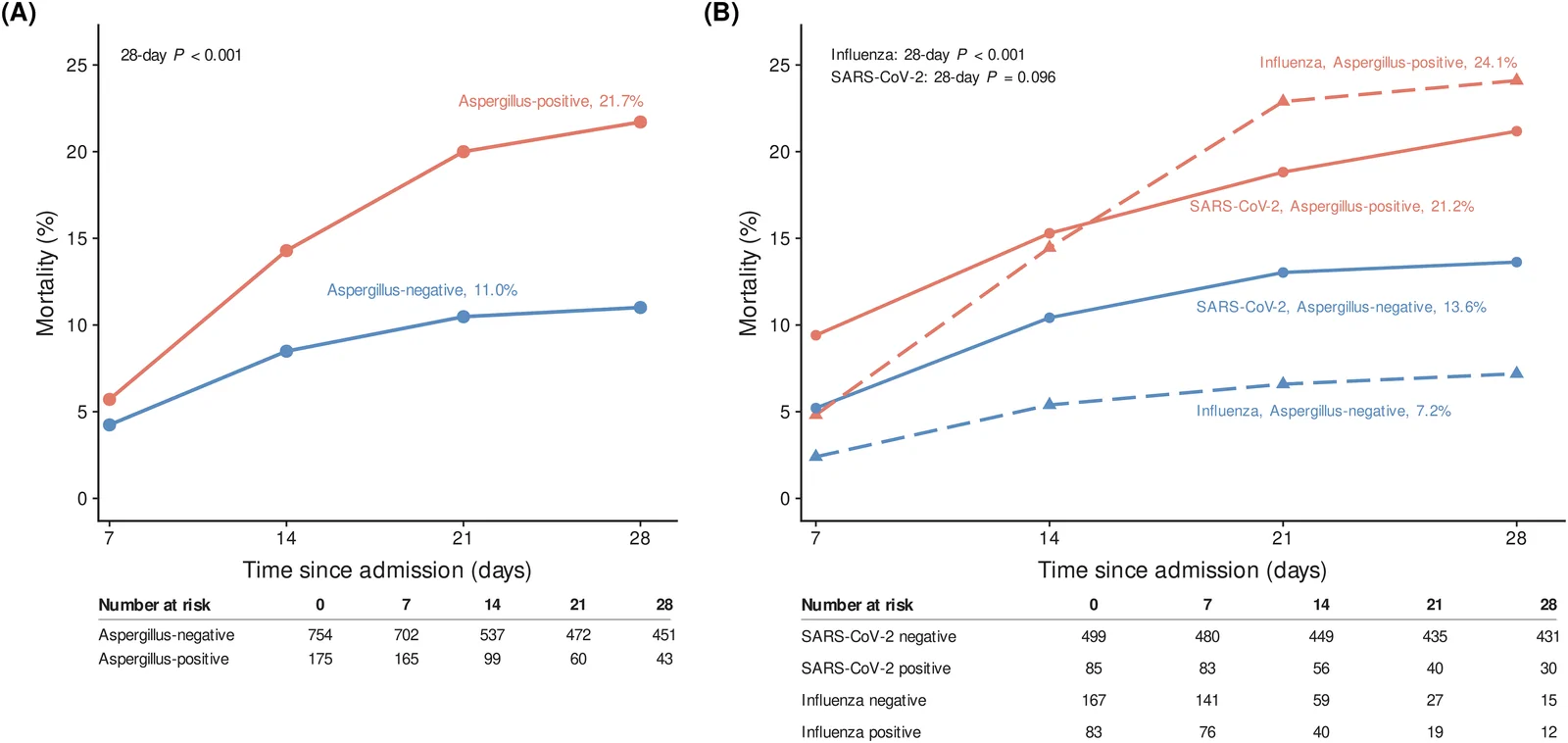
28-day in-hospital mortality among patients with viral pneumonia, with or without *Aspergillus* detection, stratified by virus type (overall, influenza, and SARS-CoV-2). **(A)** Shows 28-day in-hospital mortality among patients with viral pneumonia, with vs. without *Aspergillus* detection; **(B)** shows 28-day in-hospital mortality among influenza pneumonia and SARS-CoV-2, with vs. without *Aspergillus* detection. P values shown in the panels are two-sided and compare 28-day in-hospital mortality between *Aspergillus*-positive and *Aspergillus*-negative patients using Fisher’s exact test; in **(B)**, comparisons were performed separately within the influenza and SARS-CoV-2 subgroups.

As shown in **Table 3**, after sequential adjustment (Models 1–4), the association between *Aspergillus* detection and mortality was attenuated but remained significant [adjusted odds ratio (aOR) = 2.99, 95% CI 1.60–5.58; P < 0.001]. Among the 250 patients with influenza, mortality was 32.5% in those with detection versus 9.0% in those without; detection remained independently associated with mortality after full adjustment. Among the 584 patients with SARS-CoV-2 pneumonia, mortality was 35.3% (30/85) in those with *Aspergillus* detection compared to 14.4% (72/499) in those without. Although this association persisted across Models 1–3, it lost statistical significance after further adjustment for antiviral and antibacterial therapy, glucocorticoid use, and mechanical ventilation (Model 4). Among the 95 patients with other viral infections, mortality was 14.3% (1/7) versus 3.4% (3/88), with no independent association observed after adjustment.

In the analysis (**Supplementary Figure S3**), mechanical ventilation modified the association (P for interaction = 0.001): *Aspergillus* detection was not associated with mortality in non-ventilated patients (OR = 1.44; 95% CI 0.74–2.81; P = 0.280) but was strongly associated with mortality among ventilated patients (OR = 14.64; 95% CI 3.29–65.12; P < 0.001). Across all other subgroups, detection was consistently associated with increased mortality (ORs = 2.81–7.97, all P < 0.05), with no other significant interactions observed. While **Supplementary Figure S3** presents the overall effect modification across the entire cohort, the patterns of subgroup heterogeneity differed markedly between influenza and SARS-CoV-2 when analyzed separately (**Supplementary Figures S4**, **S5**).

### 3.4 Virus-stratified subgroup analysis of *Aspergillus* detection and mortality risk in influenza and SARS-CoV-2 pneumonia

Among patients with influenza (**Supplementary Figure S4**), the association between *Aspergillus* detection in the lower respiratory tract and mortality was consistent across sex, age, or mechanical ventilation status, with ORs ranging from 4.41 to 12.09. No statistically significant interactions were observed in all subgroups (all P > 0.05). Notably, among influenza patients receiving mechanical ventilation, mortality was 90.5% in those with *Aspergillus* detection versus 44.0% in those without (OR = 12.09; 95% CI 2.31–63.41, P = 0.003).

Among SARS-CoV-2 patients (**Supplementary Figure S5**), the association between *Aspergillus* detection and mortality showed significant subgroup heterogeneity, and the interaction with the mechanical ventilation stratification was statistically significant (interaction P-value < 0.001). In patients not receiving mechanical ventilation, *Aspergillus* detection was not significantly associated with mortality (OR = 0.54; 95% CI 0.16–1.80, P = 0.316); however, among patients receiving mechanical ventilation, the mortality rate among those with *Aspergillus* detection was 100% (27/27), and due to complete event separation, the OR could not be estimated. *Aspergillus* detection was significantly associated with mortality in male patients (OR = 3.54; 95% CI 1.95–6.42, P < 0.001) and in patients aged ≥60 years (OR = 3.09; 95% CI 1.77–5.39, P < 0.001); none of the interactions were statistically significant.

## 4 Discussion

In this multicenter cohort of 929 hospitalized patients with viral pneumonia, *Aspergillus* detection in the lower respiratory tract differed significantly by viral etiology, being highest in influenza (33.2%), intermediate in SARS-CoV-2 (14.6%), and lowest in other viral pneumonias (7.4%). *Aspergillus* detection was independently associated with a substantially increased risk of all-cause mortality, although this association varied by virus type and was most pronounced in influenza. Subgroup analysis further indicated that, in influenza pneumonia, this association remained robust across different genders, age groups, and ventilatory statuses; whereas in SARS-CoV-2 pneumonia, the impact of *Aspergillus* detection on prognosis was primarily observed in male patients, elderly patients, and those requiring mechanical ventilation, with ventilatory status showing a significant interaction (interaction P-value < 0.001).

Reported rates of viral pneumonia-associated *Aspergillus* vary widely across studies, reflecting differences in setting, case definitions, and diagnostic sensitivity rather than a single true prevalence. The pooled prevalence of IAPA in ICU patients is approximately 19.2% (range 6.4%–62.2%) (Lu et al., 2024). Similarly, the pooled prevalence of CAPA is highly heterogeneous, reported at 11.1% in ICU observational studies (Feys et al., 2021) and 17.5% in a subsequent meta-analysis (range, 2.5%–47.2%) (Gioia et al., 2024). Importantly, the burden is not confined to the ICU. CAPA has been reported in 15.7% of sub-ICU patients (Iacovelli et al., 2024) and 3.8% of inpatients during the Omicron wave (associated with a 43.5% mortality rate) (Wang et al., 2024). Furthermore, pulmonary aspergillosis occurs in 5%–33% of selected immunocompromised cohorts following other non-influenza viral infections (e.g., RSV, parainfluenza, and adenovirus) (Apostolopoulou et al., 2021).

Unlike previous studies that confined their cohorts to ICU patients or those meeting strict ECMM/ISHAM consensus criteria (Verweij et al., 2020; Koehler et al., 2021), we enrolled a broader spectrum of hospitalized adults. As a result, our endpoint captures clinically meaningful airway carriage, rather than focusing solely on adjudicated IPA. To our knowledge, no prior study has compared lower respiratory tract *Aspergillus* detection across influenza, SARS-CoV-2, and other viral pneumonias within a single cohort. Previously, the largest comparative study was confined to intubated ICU patients (Rouzé et al., 2022), while another prospective study was limited to 140 critically ill patients (Ali et al., 2022). Furthermore, other analyses examined non-influenza viruses exclusively in highly selected transplant or hematologic populations (Magira et al., 2019; Apostolopoulou et al., 2021). Therefore, this analysis provides the first integrated, virus-stratified estimate of *Aspergillus* positivity and its prognostic significance across the full severity spectrum of routine inpatient care.

The stepwise gradient in detection likely reflects virus-specific remodeling of pulmonary antifungal immunity rather than differential environmental exposure. Influenza induces a multi-level collapse of antifungal defense that includes epithelial barrier disruption, alveolar macrophage depletion, impaired phagocytic killing, and IFN-γ-driven suppression of the Th17/IL-22 axis (Short et al., 2016; Salazar et al., 2022; Feys et al., 2024; Seldeslachts et al., 2024). In a co-infection model, genetic ablation of IFN-γ restored Th17 responses and antifungal activity, identifying IFN-γ as a promising therapeutic target (Seldeslachts et al., 2024). In contrast, SARS-CoV-2 primarily causes pulmonary endotheliitis and microthrombosis (Ackermann et al., 2020; Bonaventura et al., 2021); furthermore, the associated hyperinflammation frequently requires corticosteroid therapy, thereby driving iatrogenic immunosuppression (Horby et al., 2021; Salazar et al., 2022; Shah et al., 2023; Xiang et al., 2025). Because cytokine-mediated inflammation and microvascular thrombosis are themselves dominant lethal factors in COVID-19, the independent prognostic impact of *Aspergillus* is statistically diluted by stronger mortality drivers. In our cohort, 66.3% of SARS-CoV-2 patients received glucocorticoids, compared to 23.6% of influenza patients; notably, corticosteroid exposure beyond 10 days has been linked to higher CAPA risk (OR 3.17; 95% CI 1.02–9.83) and increased mortality (77.1% vs. 43.2%, P < 0.0001) (Shah et al., 2023). These factors explain why, after adjusting for glucocorticoid use and mechanical ventilation, the prognostic significance of *Aspergillus* detection in SARS-CoV-2 patients was no longer evident (Model 4: aOR = 2.07; 95% CI 0.77–5.55; P = 0.15), and that detection of *Aspergillus* was not associated with increased mortality in SARS-CoV-2 patients not receiving mechanical ventilation (OR = 0.54, P = 0.316), suggesting that in milder cases, a positive fungal test result may represent colonization or a secondary finding rather than a lethal factor. In contrast, among mechanically ventilated patients, the 100% mortality rate in the *Aspergillus*-positive group highlighted the exceptionally poor outcomes observed in mechanically ventilated patients with severe COVID-19 and *Aspergillus* detection. However, the small sample size precluded precise risk assessment. These findings suggest that *Aspergillus* detection in COVID-19 patients primarily reflects the intensity of treatment and disease severity, rather than a direct cause of death. In infections caused by other viruses (e.g., rhinovirus, RSV) (Sajjan et al., 2008; Pickles and DeVincenzo, 2015; Horton et al., 2025), anatomically restricted airway injury, combined with younger age, lower comorbidity burden, and lower glucocorticoid exposure (16.8%), likely preserves immune reserve, thereby limiting both *Aspergillus* detection and its prognostic impact.

In viral-associated pulmonary aspergillosis, distinguishing colonization from invasive disease is challenging, as galactomannan testing and culture cannot reliably differentiate them in critically ill patients (Donnelly et al., 2019; Verweij et al., 2020). In a study utilizing histopathological validation, tissue invasion was confirmed in 55% of probable IAPA cases and 50% of probable CAPA cases, including one case without antemortem clinical suspicion (Vanderbeke et al., 2023). Others have argued that microbiological detection of *Aspergillus* in severe viral pneumonia often reflects true infection, supporting preemptive antifungal therapy in high-risk patients (Albrich and Lamoth, 2023). Our data extend these observations beyond the ICU. In this multicenter cohort encompassing multiple viral etiologies and a full spectrum of disease severity, the detection of *Aspergillus* in the lower respiratory tract was a strong and independent prognostic factor for mortality in influenza patients (fully aOR = 13.77; 95% CI 2.75–68.88) but not in those with other viral pneumonias. This is consistent with the rapid clinical course described for IAPA, with a median interval of only 3 days from ICU admission to diagnosis (Schauwvlieghe et al., 2018). Our subgroup analysis further corroborated this observation. Among mechanically ventilated influenza patients with *Aspergillus* detection, mortality reached 90.5%. This rate approaches the 90-day mortality reported for invasive tracheobronchial aspergillosis (Nyga et al., 2020). In contrast, mortality among ventilated patients without *Aspergillus* detection was 44.0%. These findings further validate this high-risk phenotype.

By evaluating lower respiratory tract *Aspergillus* detection across three viral groups under a unified framework, our study identifies “influenza plus lower respiratory tract *Aspergillus* detection” as a distinct, high-risk phenotype with an independent mortality risk several-fold higher than that of other groups. These findings challenge a uniform, one-size-fits-all approach to fungal surveillance and support a virus-stratified strategy. Given the consistent association with mortality observed across all influenza subgroups, it is recommended that adult patients hospitalized for influenza-associated pneumonia undergo testing for *Aspergillus* in the lower respiratory tract within 48 hours, regardless of gender or age. For SARS-CoV-2 pneumonia, a more nuanced strategy is warranted. Although the positive rate for routine *Aspergillus* screening may be low in patients not on mechanical ventilation, heightened vigilance is still warranted for patients requiring mechanical ventilation, particularly males and those aged 60 years and older. Testing modalities should include culture, PCR, or metagenomic next-generation sequencing, and bronchoalveolar lavage fluid galactomannan when feasible. Furthermore, preemptive antifungal therapy should be considered for any positive result. This approach aligns with recent advocacy for rapid diagnostics (Schauwvlieghe et al., 2018) and with evidence showing that empirical antifungal therapy reduces the 30-day incidence of IAPA in influenza-associated ARDS (sHR = 0.21; 95% CI 0.10–0.92), albeit without a demonstrated short-term survival benefit (Hatzl et al., 2026).

Strengths of this study include its multicenter design, the inclusion of patients across all disease severities, and the use of four sequential adjustment models to mitigate treatment-related confounding. However, several limitations must be acknowledged. First, the retrospective design precludes the elimination of unmeasured confounding and the accurate establishment of the temporal sequence between infection, detection, and treatment. Second, the study was confined to Chinese tertiary-care centers, which restricts the generalizability of the findings. Third, we lacked consensus-based classifications (e.g., AspICU, IAPA, ECMM/ISHAM) to reliably distinguish fungal colonization from invasive disease. Fourth, the limited number of events in the other-viruses subgroup (n=7) and the broad confidence intervals for influenza (e.g., OR 13.77; 95% CI 2.75-68.88) limit the statistical power of these estimates. Finally, detailed data concerning the timing, dosing, and duration of antifungal therapy were unavailable. Prospective, multicenter studies employ standardized, consensus-based definitions, larger sample sizes, and detailed data on antifungal therapy to clarify the temporal sequence between infection and treatment, reduce unmeasured confounding factors, and enhance the generalizability of our findings.

## 5 Conclusions

In this multicenter cohort, lower respiratory tract *Aspergillus* detection rates differed significantly by viral etiology, being highest in influenza, intermediate in SARS-CoV-2, and lowest in other viral pneumonias. Its prognostic significance also varied by viral etiology. *Aspergillus* detection was independently associated with in-hospital mortality in influenza pneumonia. In SARS-CoV-2 pneumonia, however, this association was attenuated after full adjustment and was primarily observed among mechanically ventilated patients. These findings support further evaluation of a virus-stratified approach to fungal surveillance and warrant prospective validation.

## Data Availability

The original contributions presented in the study are included in the article/**Supplementary Material**. Further inquiries can be directed to the corresponding authors.
