# Supplementary material for: Detection of Aspergillus in the lower respiratory tract and its association with mortality in patients with different viral pneumonias: a retrospective multicenter cohort study from China
Source: Front Cell Infect Microbiol. 2026 Sep 22;16:1906021. doi: 10.3389/fcimb.2026.1906021 (PMC13638584; doi:10.3389/fcimb.2026.1906021)
Supplement: Supplementary file 1 [file SupplementaryFile1.docx]

Supplementary Material

# 1 Supplementary Figures and Tables

## 1.1 Supplementary Figures

**Figure. S1** Distribution of virus type among patients with viral pneumonia

The bar chart illustrates the distribution of viral etiologies detected in lower respiratory tract samples from 929 patients with viral pneumonia. The majority of patients were infected with SARS-CoV-2 (n = 584, 62.9%), followed by influenza virus (n = 250, 26.9%), and other respiratory viruses (n = 95, 10.2%). Among the influenza cases, the majority were influenza A virus (Flu-A), with a small proportion of influenza B virus (Flu-B). Among all the 95 patients in the respiratory viruses, the identified viruses included ADV (n = 19), HSV (n = 19), HRV (n = 15), PIVs (n = 12), non-SARS-CoV-2 human coronavirus (n = 11), RSV (n = 10), CMV (n = 5), coxsackie (n = 2), hMPV (n = 1), and VZV (n = 1). Abbreviations: SARS-CoV-2, severe acute respiratory syndrome coronavirus 2; Flu-A, influenza A virus; Flu-B, influenza B virus; ADV, adenovirus; HSV, herpes simplex virus; HRV, human rhinovirus; PIVs, Parainfluenza viruses; RSV, respiratory syncytial virus; CMV, cytomegalovirus; hMPV, human metapneumovirus; VZV, varicella-zoster virus

**Figure. S2** Subgroup analysis of the association between viral etiology and lower respiratory tract *Aspergillus* detection

In the stratified analysis , age significantly modified this association (P for interaction = 0.008). Among patients aged ≥60 years, influenza was associated with a higher risk than SARS-CoV-2 (OR = 3.27; 95% CI 2.19–4.90; P < 0.001) while the risk in the other virus group did not differ significantly from that of SARS-CoV-2 (OR = 0.48; P = 0.167). Among those <60 years, the risk associated with influenza did not differ from that of SARS-CoV-2 (OR = 0.76; P = 0.507), but infection with other viruses was associated with a significantly lower risk (OR = 0.14; 95% CI 0.03–0.53; P = 0.004).

**Figure. S3** Subgroup analysis of *Aspergillus* detection and mortality risk in viral pneumonia

In the stratified analysis, mechanical ventilation modified the association (P for interaction = 0.001): *Aspergillus* detection was not associated with mortality in non-ventilated patients (OR = 1.44; 95% CI 0.74–2.81; P = 0.280) but was strongly associated with mortality among ventilated patients (OR = 14.64; 95% CI 3.29–65.12; P < 0.001). Across all other subgroups, detection was consistently associated with increased mortality (ORs = 2.81–7.97, all P < 0.05), with no other significant interactions observed.

**Figure. S4** Subgroup analysis of *Aspergillus* detection and mortality risk in influenza pneumonia

Among patients with influenza, the association between *Aspergillus* detection in the lower respiratory tract and mortality was consistent across sex, age, or mechanical ventilation status, with ORs ranging from 4.41 to 12.09. No statistically significant interactions were observed in all subgroups (all P > 0.05). Notably, among influenza patients receiving mechanical ventilation, mortality was 90.5% in those with *Aspergillus* detection versus 44.0% in those without (OR = 12.09; 95% CI 2.31–63.41, P = 0.003).

**Figure. S5** Subgroup analysis of Aspergillus detection and mortality risk in SARS-CoV-2 pneumonia

Among SARS-CoV-2 patients, the association between *Aspergillus* detection and mortality showed significant subgroup heterogeneity, and the interaction with the mechanical ventilation stratification was statistically significant (interaction P-value < 0.001). In patients not receiving mechanical ventilation, *Aspergillus* detection was not significantly associated with mortality (OR = 0.54; 95% CI 0.16–1.80, P = 0.316); however, among patients receiving mechanical ventilation, the mortality rate among those with *Aspergillus* detection was 100% (27/27), and due to complete event separation, the OR could not be estimated. *Aspergillus* detection was significantly associated with mortality in male patients (OR = 3.54; 95% CI 1.95–6.42, P < 0.001) and in patients aged ≥60 years (OR = 3.09; 95% CI 1.77–5.39, P < 0.001); none of the interactions were statistically significant.

## 1.2 Supplementary Tables

**Table S1 Missing data frequency of clinical variables in the study cohort**

| Variable | Miss.freq | Miss.percentage% |
| --- | --- | --- |
| Albumin (g/L) | 137 | 14.747 |
| Alanine aminotransferase (U/L) | 135 | 14.5318 |
| Aspartate aminotransferase (U/L) | 156 | 16.7922 |
| Creatine kinase | 182 | 19.591 |
| Serum creatinine | 8 | 0.8611 |
| CRP, mg/L | 105 | 11.3025 |
| D-dimer (ng/mL) | 158 | 17.0075 |
| Diastolic blood pressure, mmHg | 129 | 13.8859 |
| Hemoglobin (g/L) | 5 | 0.5382 |
| Lac., mmol/L | 257 | 27.6642 |
| LDH, U/L | 172 | 18.5145 |
| Lymphocyte count (×10⁹/L) | 5 | 0.5382 |
| Monocyte count (×10⁹/L) | 133 | 14.3165 |
| Neutrophil count (×10⁹/L) | 7 | 0.7535 |
| Oxygenation index (mmHg) | 251 | 27.0183 |
| Pulse rate (bpm) | 128 | 13.7783 |
| PaCO₂, (mmHg) | 232 | 24.9731 |
| PaO₂ (mmHg) | 242 | 26.0495 |
| PCT (ng/mL) | 69 | 7.4273 |
| Arterial pH | 243 | 26.1572 |
| Platelet count (×10⁹/L) | 130 | 13.9935 |
| prothrombin time (s) | 183 | 19.6986 |
| Respiratory rate (bpm) | 129 | 13.8859 |
| Systolic blood pressure (mmHg) | 129 | 13.8859 |
| Temperature (Tmax,°C) | 128 | 13.7783 |
| WBC (×10⁹/L) | 8 | 0.8611 |

Data are presented as number of missing values (Miss.freq) and percentage of missing values (Miss.percentage%). Miss.percentage% was calculated as (Miss.freq / 929) × 100%. Abbreviations: CRP, C-reactive protein; Lac., lactate; LDH, lactate dehydrogenase; PCT, procalcitonin; WBC, white blood cell count; PaCO₂, arterial partial pressure of carbon dioxide; PaO₂, arterial partial pressure of oxygen

**Table S2** Baseline characteristics of patients with different types of viral pneumonia

| **Variables** | **Total (n = 929)** | **SARS-Cov-2 (n = 584)** | **Flu (n = 250)** | **Other viruses (n = 95)** | **p** | **Statistic** |
| --- | --- | --- | --- | --- | --- | --- |
| **Physical examination** | | | | | | |
| Temperature (Tmax), °C, Mean ± SD | 37.2 ± 1.0 | 37.0 ± 0.8 | 37.4 ± 1.1 | 38.0 ± 1.3 | < 0.001 | 53.246 |
| Pulse rate (bpm), Mean ± SD | 90.7 ± 22.2 | 91.3 ± 24.6 | 90.2 ± 18.9 | 88.6 ± 13.3 | 0.507 | 0.68 |
| Systolic blood pressure (mmHg), Mean ± SD | 131.8 ± 20.1 | 133.9 ± 20.0 | 128.8 ± 20.9 | 126.4 ± 16.9 | < 0.001 | 9.768 |
| Diastolic blood pressure (mmHg), Mean ± SD | 77.6 ± 12.6 | 78.4 ± 12.5 | 76.0 ± 13.3 | 76.7 ± 11.0 | 0.033 | 3.434 |
| Respiratory rate (bpm), Mean ± SD | 21.4 ± 4.0 | 21.3 ± 3.7 | 21.9 ± 4.8 | 21.4 ± 3.8 | 0.116 | 2.16 |
| **Laboratory tests** | | | | | | |
| WBC count (×10⁹/L), Mean ± SD | 8.4 ± 5.3 | 8.2 ± 5.0 | 8.5 ± 5.4 | 9.2 ± 6.4 | 0.197 | 1.626 |
| Neutrophil count (×10⁹/L), Median (IQR) | 5.3 (3.4, 8.7) | 5.3 (3.5, 8.5) | 5.2 (3.3, 8.8) | 5.3 (3.6, 8.6) | 0.933 | 0.138 |
| Lymphocyte count (×10⁹/L), Median (IQR) | 0.9 (0.5, 1.3) | 0.8 (0.5, 1.2) | 0.8 (0.6, 1.4) | 1.3 (0.9, 1.8) | < 0.001 | 48.866 |
| Monocyte count (×10⁹/L), Median (IQR) | 0.5 (0.3, 0.7) | 0.5 (0.3, 0.8) | 0.5 (0.3, 0.7) | 0.6 (0.3, 0.7) | 0.529 | 1.275 |
| Hemoglobin (g/L), Mean ± SD | 122.4 ± 22.9 | 121.7 ± 23.3 | 122.2 ± 22.4 | 126.9 ± 21.7 | 0.128 | 2.06 |
| Platelet count (×10⁹/L), Mean ± SD | 210.4 ± 96.8 | 212.0 ± 95.7 | 205.7 ± 93.5 | 213.2 ± 111.6 | 0.665 | 0.408 |
| Alanine aminotransferase (U/L), Median (IQR) | 24.0 (16.0, 38.0) | 23.0 (15.0, 36.0) | 24.0 (16.0, 46.0) | 24.0 (17.0, 34.0) | 0.041 | 6.397 |
| Aspartate aminotransferase (U/L), Median (IQR) | 31.0 (20.0, 51.0) | 33.0 (21.0, 56.0) | 30.0 (19.0, 49.0) | 24.0 (18.5, 35.5) | < 0.001 | 17.23 |
| LDH (U/L), Median (IQR) | 250.0 (200.0, 336.0) | 256.0 (204.0, 332.0) | 248.0 (199.0, 344.5) | 226.0 (188.0, 322.0) | 0.163 | 3.634 |
| Creatine kinase (U/L), Median (IQR) | 86.0 (47.0, 180.0) | 97.0 (49.0, 193.2) | 75.5 (42.5, 174.0) | 65.0 (43.0, 125.5) | 0.009 | 9.512 |
| Serum creatinine (μmol/L), Median (IQR) | 72.0 (58.0, 94.0) | 77.0 (62.0, 103.0) | 66.0 (53.0, 82.0) | 65.0 (54.5, 80.0) | < 0.001 | 43.293 |
| PT (s), Median (IQR) | 12.4 (11.6, 13.2) | 12.4 (11.6, 13.3) | 12.3 (11.5, 13.1) | 12.3 (11.6, 13.1) | 0.58 | 1.091 |
| CRP (mg/L), Median (IQR) | 41.0 (13.0, 96.5) | 38.8 (12.2, 92.8) | 46.8 (17.2, 104.6) | 41.2 (9.7, 117.6) | 0.268 | 2.634 |
| PCT (ng/mL), Median (IQR) | 0.1 (0.1, 0.5) | 0.1 (0.1, 0.5) | 0.2 (0.1, 0.6) | 0.1 (0.0, 0.5) | 0.038 | 6.544 |
| pH, Mean ± SD | 7.4 ± 0.1 | 7.4 ± 0.1 | 7.4 ± 0.1 | 7.4 ± 0.1 | 0.007 | 5.011 |
| PaO₂ (mmHg), Mean ± SD | 87.2 ± 34.5 | 87.8 ± 35.2 | 87.5 ± 35.2 | 82.5 ± 27.4 | 0.374 | 0.984 |
| Oxygenation index (mmHg), Mean ± SD | 356.6 ± 157.0 | 368.8 ± 165.5 | 337.3 ± 137.7 | 333.1 ± 144.2 | 0.009 | 4.739 |
| PaCO₂ (mmHg), Mean ± SD | 37.7 ± 10.6 | 37.2 ± 10.8 | 38.4 ± 9.8 | 38.8 ± 11.8 | 0.177 | 1.734 |
| Lactic acid (mmol/L), Median (IQR) | 1.5 (1.1, 2.0) | 1.6 (1.2, 2.2) | 1.4 (1.0, 1.9) | 1.4 (1.1, 1.7) | < 0.001 | 30.051 |

Data are presented as n (%), mean ± SD, or median (IQR). Comparisons among groups were performed using one-way analysis of variance (ANOVA) for normally distributed variables, Kruskal–Wallis test for non-normally distributed variables, and chi-square test for categorical variables. Abbreviations: WBC, white blood cell; AST, aspartate aminotransferase; LDH, lactate dehydrogenase; PT, prothrombin time; PaO₂, arterial partial pressure of oxygen; PaCO₂, arterial partial pressure of carbon dioxide; CRP, C-reactive protein; PCT, procalcitonin

**Table S3** Univariate logistic regression analysis of factors associated with *Aspergillus* detection in patients with viral pneumonia

| **Variable** | **OR (95% CI)** | **P value** |
| --- | --- | --- |
| **Demographic and clinical characteristics** |  |  |
| Age | 0.98 (0.97–0.99) | <0.001 |
| Male | 0.90 (0.64–1.27) | 0.561 |
| Smoking history | 0.45 (0.30–0.69) | <0.001 |
| Length of hospital stay | 0.98 (0.97–1.00) | 0.046 |
| T_max_ (°C) | 1.13 (0.97–1.31) | 0.125 |
| Pulse rate (bpm) | 1.01 (1.00–1.01) | 0.014 |
| Respiratory rate (breaths/min) | 1.03 (1.00–1.07) | 0.081 |
| Systolic blood pressure (mmHg) | 1.00 (0.99–1.01) | 0.495 |
| Diastolic blood pressure (mmHg) | 1.00 (0.99–1.01) | 0.802 |
| **Laboratory tests** |  |  |
| WBC count (×10⁹/L) | 1.05 (1.02–1.07) | 0.002 |
| Neutrophil count (×10⁹/L) | 1.05 (1.02–1.09) | 0.001 |
| Lymphocyte count (×10⁹/L) | 1.02 (0.98–1.06) | 0.25 |
| Monocyte count (×10⁹/L) | 0.94 (0.74–1.19) | 0.602 |
| Hemoglobin (g/L) | 1.00 (0.99–1.01) | 0.926 |
| Platelet count (×10⁹/L) | 1.00 (1.00–1.00) | 0.735 |
| ALT (U/L) | 1.00 (0.99–1.00) | 0.895 |
| AST (U/L) | 1.00 (1.00–1.00) | 0.784 |
| LDH (U/L) | 1.00 (1.00–1.00) | 0.101 |
| Creatine kinase (U/L) | 1.00 (1.00–1.00) | 0.719 |
| Albumin (g/L) | 0.99 (0.96–1.01) | 0.35 |
| Serum creatinine (μmol/L) | 1.00 (1.00–1.00) | 0.318 |
| PT (s) | 0.99 (0.97–1.02) | 0.529 |
| D-dimer (ng/mL) | 1.00 (1.00–1.00) | 0.001 |
| CRP (mg/L) | 1.00 (1.00–1.00) | 0.245 |
| PCT (ng/mL) | 1.01 (1.00–1.02) | 0.087 |
| Arterial pH | 0.69 (0.11–4.27) | 0.685 |
| PaO₂ (mmHg) | 1.00 (0.99–1.00) | 0.888 |
| PaCO₂ (mmHg) | 1.01 (0.99–1.02) | 0.509 |
| Oxygenation index (mmHg) | 1.00 (1.00–1.00) | 0.113 |
| Lactic acid (mmol/L) | 0.96 (0.84–1.10) | 0.596 |
| **Comorbidities** |  |  |
| Hypertension | 0.90 (0.65–1.25) | 0.53 |
| Coronary heart disease | 2.40 (1.69–3.40) | <0.001 |
| Diabetes mellitus | 1.32 (0.93–1.88) | 0.125 |
| Chronic lung diseases | 3.29 (2.30–4.71) | <0.001 |
| Chronic kidney disease | 1.52 (0.86–2.66) | 0.147 |
| Cerebrovascular disease | 0.93 (0.55–1.58) | 0.798 |
| Chronic liver disease | 2.25 (1.16–4.38) | 0.016 |
| Immunosuppression | 1.13 (0.70–1.83) | 0.62 |
| **Treatment** |  |  |
| Antiviral treatment | 5.28 (3.44–8.10) | <0.001 |
| Antibacterial treatment | 7.00 (2.19–22.40) | 0.001 |
| Glucocorticoid treatment | 1.67 (1.19–2.33) | 0.003 |
| Mechanical ventilation | 3.58 (2.37–5.40) | <0.001 |
| **Virus type (ref: SARS-CoV-2)** |  |  |
| Influenza virus | 2.92 (2.06–4.14) | <0.001 |
| Other viruses | 0.47 (0.21–1.04) | 0.063 |

Abbreviations: OR, odds ratio; CI, confidence interval; T_max_, maximum temperature; WBC, white blood cell; ALT, alanine aminotransferase; AST, aspartate aminotransferase; LDH, lactate dehydrogenase; PT, prothrombin time; PaO₂, partial pressure of arterial oxygen; PaCO₂, partial pressure of arterial carbon dioxide; CRP, C-reactive protein; PCT, procalcitonin. Virus type with SARS-CoV-2 as the reference group
